# Modulation of tumor-associated lymphangiogenesis by combination immunotherapy approaches in triple-negative breast cancer: a systematic review

**DOI:** 10.3389/fimmu.2026.1641259

**Published:** 2026-04-21

**Authors:** María Guerrero-Sánchez, Lisa Martens, Melissa García-Caballero

**Affiliations:** 1 Biomedical Research Institute of Málaga and Nanotechnology Platform (IBIMA-Plataforma BIONAND), Málaga, Spain; 2 Faculty of Medicine and Life Sciences, Hasselt University, Diepenbeek, Belgium; 3 Department of Molecular Biology and Biochemistry, Faculty of Sciences, University of Málaga, Málaga, Spain

**Keywords:** immunotherapy, lymphatic endothelial cell, target, triple negative breast cancer, tumor-associated lymphangiogenesis

## Abstract

**Background:**

Treatment for triple-negative breast cancer (TNBC) is challenging due to its aggressive nature and its high metastatic potential. This breast cancer subtype is characterized by the presence of highly proliferative cells with great ability to promote lymphangiogenesis and facilitate metastasis to regional lymph nodes (LNs). Indeed, the physical presence of breast cancer cells in regional LNs is an important prognostic factor. Moreover, tumor-associated lymphangiogenesis within the TNBC microenvironment plays a crucial role not only providing new routes for the metastatic spread of cancer cells but also delineating tumor immunity. As such, modulating tumor-associated vasculature by combination immunotherapy could be a promising strategy to treat TNBC.

**Methods:**

In this study, we have conducted a comprehensive review of previously published works using PubMed and Web of Science databases for studies addressing immunotherapy and lymphangiogenesis in TNBC. Inclusion criteria were applied to identify relevant preclinical and clinical studies focusing on the intersection of immune-based therapies and the tumor lymphatic network. A total of 59 eligible articles were identified and analyzed.

**Results:**

Our findings indicate that, while immunotherapy is an effective clinical strategy to treat TNBC, current strategies rarely incorporate agents targeting tumor-associated lymphangiogenesis. The reviewed studies suggest that lymphatic vessels within the TNBC microenvironment are not only structural pathways for metastasis but also dynamic regulators of immune cell trafficking and tumor immune evasion. Despite this, the therapeutic potential of combining immunotherapy with lymphangiogenesis-targeting agents remains underexplored.

**Conclusion:**

Despite clear evidence supporting the role of lymphangiogenesis in TNBC progression and immune regulation, its integration into immunotherapy regimens remains largely unexplored. Therefore, the development of novel combination strategies based on immunotherapy and agents targeting lymphatic vessels and their crosstalk with immune and cancer cells within the TNBC microenvironment would greatly improve the response of TNBC patients to immunotherapy.

## 1 Introduction

Cancer is considered one of the major societal, health, and economic problems in the 21^st^ century. Among the different cancer types, breast cancer was the second most commonly occurring cancer worldwide in 2022, with 2.3 million new cases, accounting for 11.6% of the total of cancer cases diagnosed (1). It is a genetically and clinically heterogeneous disease with distinct subtypes commonly categorized into four main groups based on the immunohistochemical expression of hormone receptors: estrogen receptor positive (ER+), progesterone receptor positive (PR+), human epidermal growth factor receptor positive (HER2+), and triple-negative (TNBC), which is characterized by the lack of expression of any of the above receptors (2).

TNBC mostly occurs in premenopausal young women under 40 years old, comprising approximately 15–20% of all breast cancer patients (3). The risk of developing TNBC varies with genetics, race, age, overweight and obesity, breastfeeding patterns, and parity, with genetic factors, particularly BRCA1/2 mutations (4), manifesting the highest contribution (5). TNBC is known for its high molecular heterogeneity, metastatic potential, and poor prognosis (5). It is a very aggressive tumor subtype, characterized by highly proliferative cells with great spread capacity (2, 3, 6–8). Although in recent years important progress has been made in breast cancer prevention and treatment, effective therapies against the TNBC subtype are still limited. Due to the absence of target receptors (ER, PR, and HER2), specific therapies targeted against these proteins are useless in this subtype, limiting the treatment options (7, 9). In addition, TNBC has high recurrence and distant metastasis rates, that further complicates finding an effective clinical alternative. In this scenario, chemotherapy remains the standard of care for TNBC patients, although frequently they develop resistance, significant side effects, and treatment efficacy varies between patients (9, 10). Therefore, identifying new therapeutic approaches for the treatment of the TNBC is crucial to overcome the current limitations associated with the disease management.

The tumor microenvironment (TME), comprising the cancerous and non-cancerous host cells in the tumor (fibroblasts, endothelial cells, neurons, adipocytes, and immune cells, as well as non-cellular components, including the extracellular matrix and signaling molecules), is crucial in determining tumor progression and response to treatment (11–14). In the TNBC microenvironment, lymphatic vessels are essential components and play a central role in fluid homeostasis, immune cell trafficking, and metastatic dissemination (15–18). Structurally, lymphatic capillaries are characterized by a discontinuous or absent basement membrane, loosely connected endothelial cell junctions, and anchoring filaments that facilitate interstitial fluid uptake, distinguishing them from blood vessels and enabling efficient transport of cells and macromolecules. Lymphatic vessel formation, named as lymphangiogenesis, is a tightly regulated process driven by multiple molecular signals. While the VEGF-C/VEGFR-3 axis represents the central pathway controlling LEC proliferation and migration, additional factors such as VEGF-D, angiopoietins (ANGPT1/2), fibroblast growth factors (FGFs), platelet-derived growth factor (PDGF-B), inflammatory cytokines (e.g., IL-6, TNF-α), hypoxia-associated signaling, and cytokines derived from tumor and stromal cells also contribute to lymphatic remodeling. These pathways collectively contribute to lymphatic vessel expansion, functional remodeling within the TME and cancer cell spread to regional lymph nodes (LNs) and distant sites (15–18). Importantly, targeting these signaling axes may provide complementary therapeutic strategies to inhibit tumor-associated lymphangiogenesis.

Metastasis in TNBC patients occurs preferentially through the lymphatic vasculature, being more frequent this dissemination when compared to the blood vessel-mediated spreading (19). On the other hand, the lymphatic system can regulate tumor immunity by directly regulating immune cell entry and migration or, indirectly by modulating which antigens and cells are delivered to regional LNs through the regulation of lymphatic vessel tone and pumping (20, 21). Thus, the strong association between tumor-induced lymphangiogenesis and the immune system in the TME highlights the key role of lymphatics in tumor progression and the impact of targeting lymphatics as a promising approach to treat TNBC.

TNBC has the highest immunogenic potential and proportion of tumor infiltrating lymphocytes (TILs) of all subtypes of breast cancer, making immunotherapy an interesting therapeutic option against this tumor subtype (22–24). A high density of TILs indicates that T-cells are already present in the TME and that can be reactivated by immunotherapy. The immune system of cancer patients recognizes tumor-specific molecules enabling it to attack cancer cells (25, 26), and in turn, tumor cells can express a variety of checkpoint proteins to evade or suppress the patient’s immune response. Currently, the most successful immunotherapeutic agents for TNBC are immune-checkpoint inhibitors that block immunosuppressive receptors expressed on tumor cells, such as programmed death-ligand 1/programmed death 1 (PD-L1/PD-1), stimulating anti-tumor immune responses and promoting the elimination of tumor cells (26–28). However, not all patients respond equally well to this treatment, mainly because not everyone has high PD-L1 levels. To improve immunotherapy success, we propose the use of combination immunotherapy approaches, which refer to the application of immunotherapies with other traditional or conventional modalities used in the treatment of cancer (i.e. radiotherapy, chemotherapy, targeted therapies, etc.) to potentially enhance its efficacy with diminished toxic effects. In this context, combining immunotherapies with agents targeting tumor lymphatics seems a promising strategy.

Overcoming the limitations of current TNBC treatments requires innovative approaches that go beyond traditional strategies. Integrating immunotherapy with agents targeting tumor-associated lymphangiogenesis could present an opportunity to improve the therapeutic landscape for this cancer subtype, as it has the potential to effectively disrupt the core mechanisms facilitating tumor cell migration and metastatic spread through the lymphatic system. Accordingly, this systematic review aims at analyzing the published information on TNBC treatments, with special focus on immunotherapy, and tumor-associated lymphangiogenesis, emphasizing the benefits of novel strategies modulating the immune compartment and the tumor lymphatics for combating TNBC.

## 2 Methods

**Figure 1:**
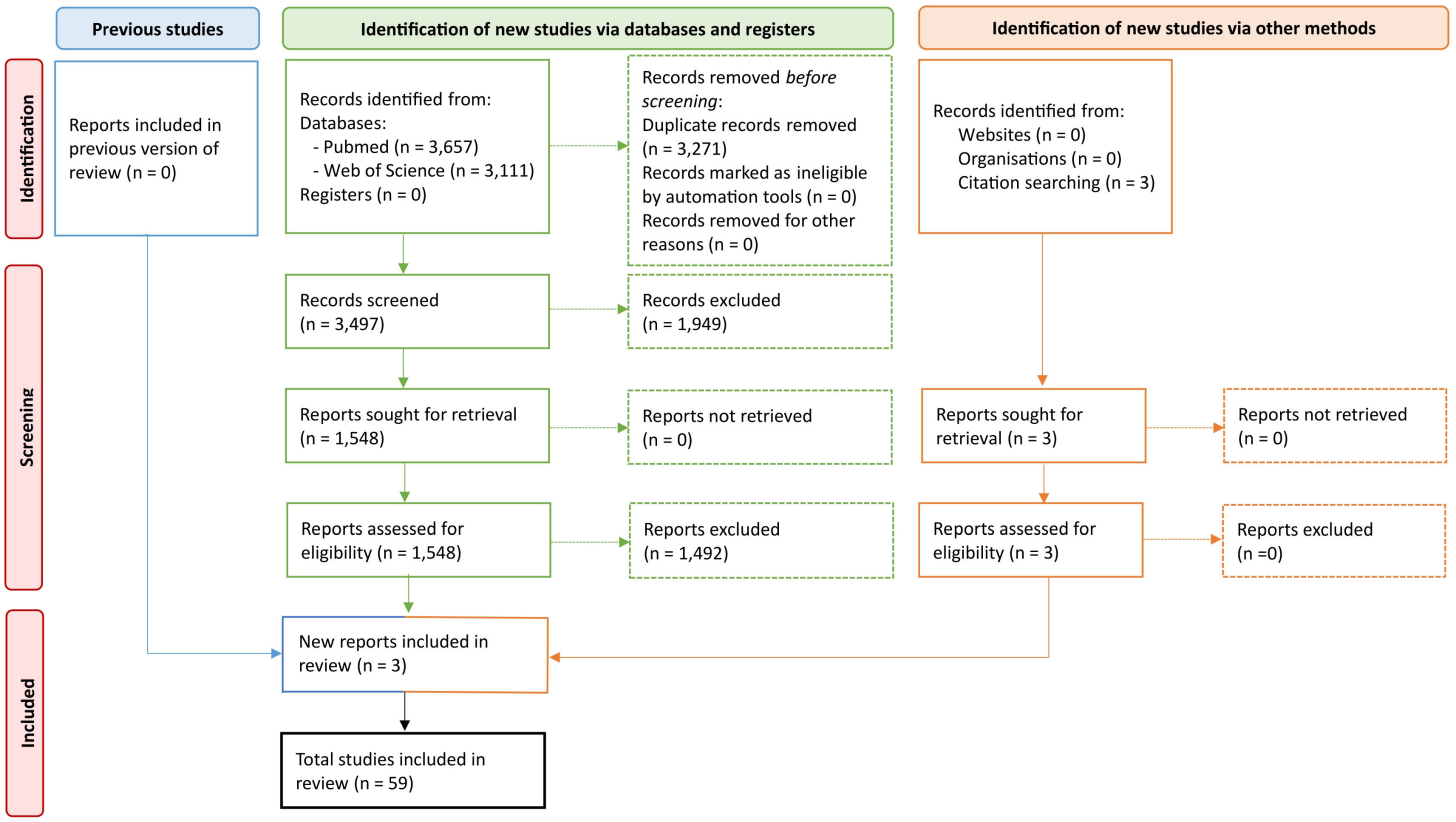
PRISMA flow diagram of study selection. This figure illustrates the PRISMA (Preferred Reporting Items for Systematic Reviews and Meta-Analyses) flow diagram showing the study selection process. The diagram includes the number of records identified, screened, assessed for eligibility, and included in the systematic review.

A systematic literature search was performed on two major electronic databases: PubMed and Web of Science, to investigate the possible role of targeting tumor-associated lymphangiogenesis in a combination immunotherapy treatment for TNBC. The search encompassed all articles published up to April 19, 2025, ensuring a comprehensive dataset that captured the most recent advancements in the field. In addition to the database search, a manual review of the reference lists from selected studies was performed to identify potentially relevant articles that may not have been captured through the initial search. This dual approach ensured a robust and exhaustive collection of studies. The search process adhered to the PRISMA (Preferred Reporting Items for Systematic Reviews and Meta-Analyses) guidelines (29). The detailed methodology is outlined in a PRISMA flowchart provided in **Figure 1**. Identified articles were de-duplicated using Endnote software, after which the unique entries were imported into Rayyan, a centralized platform designed for systematic reviews, to facilitate the screening process.

To ensure the inclusion of only relevant studies, titles and abstracts were independently reviewed by two of the authors (MG-S and MG-C), followed by a detailed full-text evaluation conducted by MGS for potentially eligible articles. Inclusion and exclusion criteria were pre-defined between the authors (MG-S and MG-C) to standardize the selection process. Studies were included if they explicitly explored the intersection of lymphangiogenesis, immunotherapy, and TNBC. In addition, exclusion criteria were applied rigorously, excluding gray literature (e.g., conference abstracts, proceedings, and editorials), review articles, and non-English publications.

The search strategy was carefully designed to encompass a range of terms associated with lymphangiogenesis and immunotherapy in TNBC. Therefore, the keywords included were “lymphangiogenesis”, “immunotherapy”, and “triple-negative breast cancer”. Additionally, alternative terms were incorporated to further refine the search: “agent”, “block”, “drug”, “model”, “modulate”, and “target”. Multiple search queries were employed to maximize the breadth of retrieved articles, combining these terms with Boolean operators such as “AND” to ensure specificity and comprehensiveness. The exact queries used to obtain the data from the two databases were as follows: “lymphangiogenesis AND immunotherapy AND triple-negative breast cancer”, “lymphangiogenesis AND immunotherapy”, “lymphangiogenesis AND triple-negative breast cancer AND agent”, “lymphangiogenesis AND triple-negative breast cancer AND drug”, “lymphangiogenesis AND triple-negative breast cancer AND model”, “lymphangiogenesis AND triple-negative breast cancer AND target”, “immunotherapy AND triple-negative breast cancer AND agent”, “immunotherapy AND triple-negative breast cancer AND block”, “immunotherapy AND triple-negative breast cancer AND drug”, “immunotherapy AND triple-negative breast cancer AND modulate”, and “immunotherapy AND triple-negative breast cancer AND target”.

This precise approach was designed to capture studies that elucidate the mechanisms through which lymphangiogenesis can be modulated and integrated with combination immunotherapy as an innovative therapeutic strategy for TNBC treatment. The systematic identification and selection of articles aimed to provide a solid foundation for understanding current trends, knowledge gaps, and potential directions for future research in this promising field.

## 3 Results

Our comprehensive database search initially resulted in a total of 6,768 studies. Following the removal of duplicate records, 3,497 unique articles were retained for further evaluation. Titles and abstracts were screened, resulting in 1,548 articles eligible for a more in-depth review. After screening of the full-texts, a final set of 59 articles was included and evaluated according to the selection criteria predefined by the authors (**Figure 1**).

### 3.1 Current therapies approved for the treatment of triple-negative breast cancer

In recent years, therapies approved by the U.S. Food and Drug Administration (FDA) for the treatment of TNBC have expanded considerably beyond traditional chemotherapy approaches (**Table 1**). While conventional chemotherapeutic agents such as Paclitaxel, Doxorubicin, Cyclophosphamide, and Carboplatin remain essential components of treatment regimens and constitute the backbone of TNBC therapy (9, 10), these agents have not been approved specifically for the TNBC as a distinct molecular subtype. Instead, they are approved for the treatment of breast cancer more broadly and are widely incorporated into TNBC treatment protocols. In this context, chemotherapy is frequently used as part of FDA-approved combination strategies, particularly in combination with immunotherapy. Notably, combinations such as Pembrolizumab plus chemotherapy and Atezolizumab plus Nab-paclitaxel have demonstrated clinical benefit in selected TNBC patient populations (30, 31). It is important to note that although Atezolizumab plus Nab-paclitaxel initially received accelerated approval for PD-L1-positive metastatic TNBC, this indication was later withdrawn. However, it remains highly relevant in the clinical and scientific literature. Moreover, the lack of estrogen, progesterone, and HER2 receptor expression in TNBC limits the use of endocrine and HER2-targeted therapies, thereby restricting treatment options and contributing to the clinical challenges associated with this subtype (2, 7, 9).

**Table 1:** FDA approved therapies for the treatment of triple-negative breast cancer.

Agent	Approval date	Brand name/manufacturer	Indication
Olaparib	January 12, 2018	Lynparza/AstraZeneca Pharmaceuticals LP	Locally advanced or metastatic HER2-negative breast cancer with germline BRCA mutation previously treated with chemotherapy
Talazoparib	October 16, 2018	Talzenna/ Pfizer Inc.	Locally advanced or metastatic HER2-negative breast cancer with germline BRCA mutation
Atezolizumab + Nab-paclitaxel	March 8, 2019	Tecentriq/Genentech Inc.	Adult with unresectable locally advanced or metastatic TNBC whose tumors express PD-L1 ≥1% on immune cells
Pembrolizumab + chemotherapy	November 13, 2020	Keytruda/ Merck & Co.	Locally recurrent unresectable or metastatic TNBC with PD-L1 expression (CPS ≥10)
Sacituzumab govitecan	April 22, 2020 (accel.); April 7, 2021 (full)	Trodelvy/Immunomedics Inc.	Unresectable locally advanced or metastatic TNBC who received ≥2 prior therapies (including ≥1 for metastasis)
Pembrolizumab (neoadjuvant and adjuvant)	July 26, 2021	Keytruda/ Merck	High-risk early-stage TNBC in combination with chemotherapy (neoadjuvant) and as monotherapy post-surgery (adjuvant)

Data extracted from the FDA database.

To address this challenge, several targeted and immunotherapeutic strategies have been developed and approved for specific subgroups of patients with TNBC. Among them, Poly (ADP-ribose) polymerase (PARP) inhibitors such as Olaparib and Talazoparib, both approved in 2018, are indicated for patients with locally advanced or metastatic HER2-negative breast cancer with germline BRCA-mutation (32, 33).

The following year Atezolizumab in combination with Nab-paclitaxel was approved for adults with unresectable locally advanced or metastatic TNBC whose tumors express PD-L1 ≥ 1% on immune cells (30). This was closely followed by the 2020 approval of Pembrolizumab in combination with chemotherapy for patients with recurrent or metastatic PD-L1-positive TNBC, defined by a combined positive score (CPS) ≥ 10 (31). These approvals underscore the growing role of immune checkpoint inhibitors in treating PD-L1-positive metastatic TNBC (22).

Additionally, Sacituzumab govitecan, an antibody-drug conjugate targeting Trop-2, received accelerated approval in 2020 and full approval in 2021 for patients with locally advanced or metastatic TNBC who had received two or more prior therapies (34). Most recently, in July 2021, Pembrolizumab was approved for use in both neoadjuvant and adjuvant settings for patients with high-risk early-stage TNBC, representing a pivotal shift toward incorporating immunotherapy in earlier stages of the disease (35).

Despite these advances, the pronounced heterogeneity and aggressive behavior of TNBC continue to limit long-term treatment success, highlighting the urgent need to develop novel and more precise therapeutic approaches.

### 3.2 Monotherapy and combination strategies in evaluation for triple-negative breast cancer

**Figure 2:**
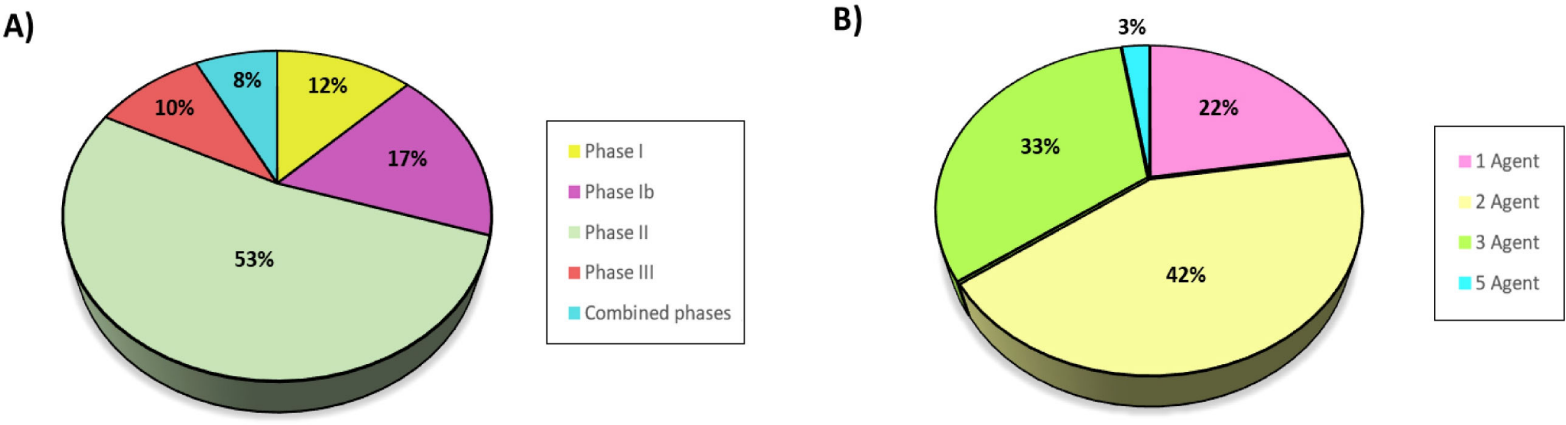
Number of triple-negative breast cancer clinical trials by **(a)** study phase and **(b)** therapeutic agents used per trial.

We identified a total of 40 clinical trials that studied the efficacy of various therapeutic strategies and agents for TNBC. The majority of these trials were conducted in study phase II (21 studies), followed by phase Ib (7 studies), phase I (5 studies), and phase III (4 studies). Additionally, three trials combined two phases in their design (**Figure 2a**). In terms of treatment strategies, we observed that 9 trials employed monotherapies, while 31 explored combination therapies. Among the combination strategies, the most frequently studied were those of two-agent, reported in 17 studies, followed by three-agent, presented in 13 studies. Notably, one trial investigated a five-agent combination (**Figure 2b**). Interestingly, although a wide range of drug combinations has been reported, only two specific combinations have been consistently investigated: Atezolizumab plus Nab-paclitaxel and Durvalumab plus Nab-paclitaxel. Both Atezolizumab and Durvalumab function as PD-L1 inhibitors, enhancing the immune system’s ability to recognize and attack tumor cells, while Nab-paclitaxel is a chemotherapy agent that disrupts cancer cell proliferation. These drug combinations demonstrated significant clinical benefit, including improvements in terms of overall survival (OS), progression-free survival (PFS), total pathological complete response (pCR), and tumor response. The positive outcomes were particularly pronounced in patients with advanced or metastatic TNBC (36–40).

**Figure 3:**
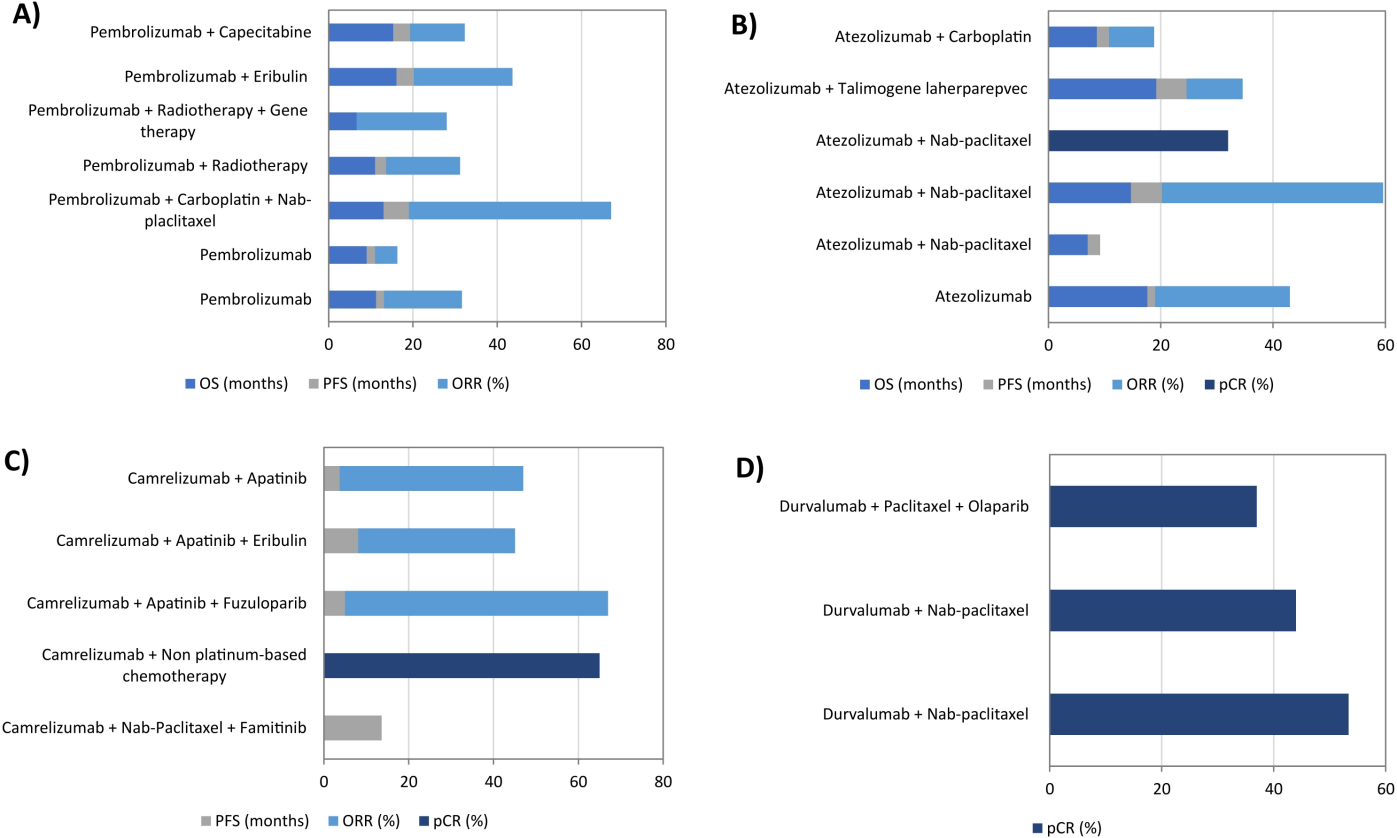
Effect of the four most commonly used immunotherapeutic agents on the treatment of triple-negative breast cancer. **(a)** Pembrolizumab; **(b)** Atezolizumab; **(c)** Camrelizumab; **(d)** Durvalumab. pCR, total pathological complete responses; PFS, progression-free survival; ORR, overall response rate; OS, overall survival.

**Figure 4:**
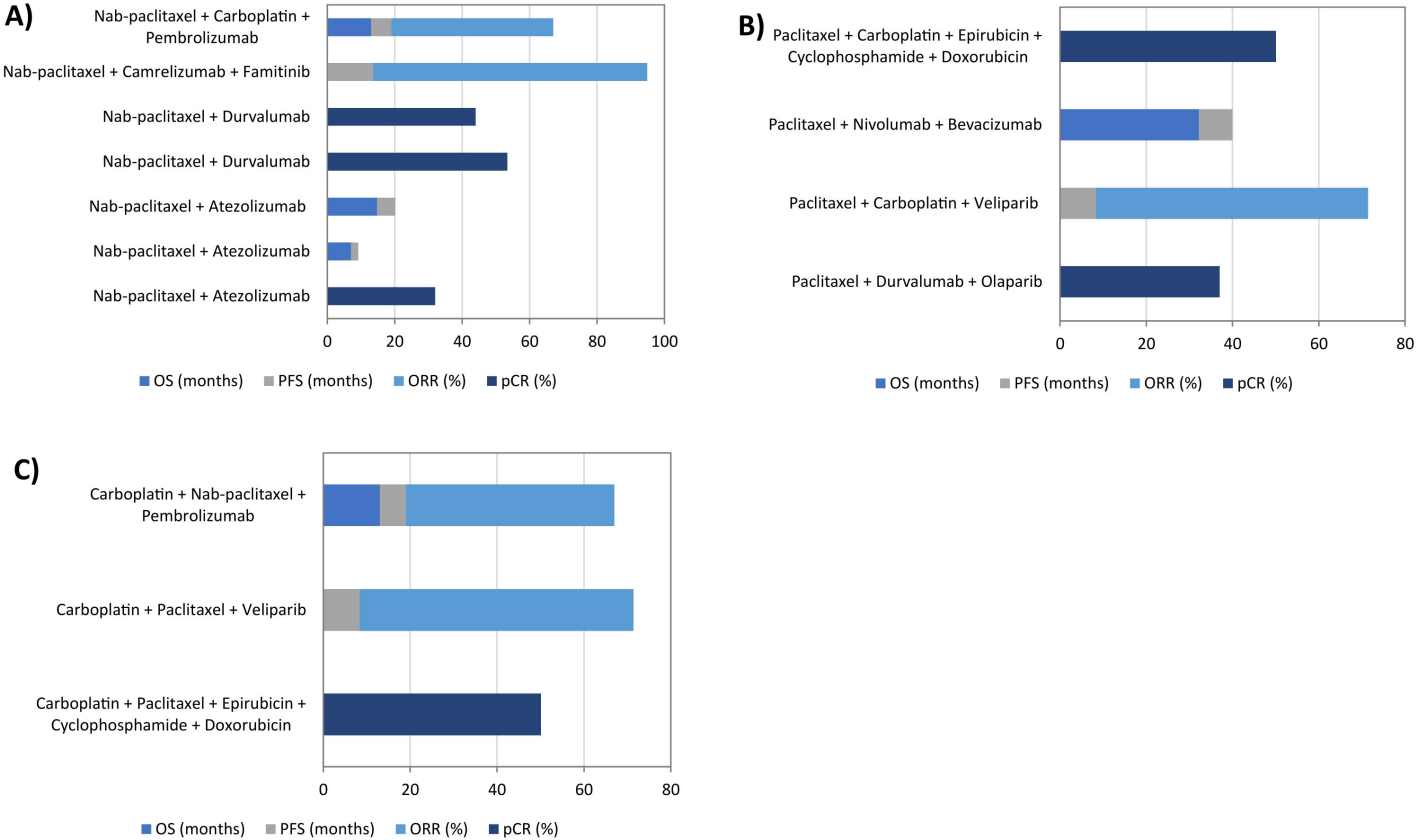
Effect of the three most commonly used chemotherapeutic agents on the treatment of triple-negative breast cancer. **(a)** Nab-paclitaxel; **(b)** Paclitaxel; **(c)** Carboplatin. pCR: total pathological complete responses; PFS: progression-free survival; ORR, overall response rate; OS, overall survival.

Regarding the agents analyzed in the clinical trials, the effects of a total of 42 different drugs were reported. These drugs were systematically classified into four major groups: immunotherapy, chemotherapy, targeted therapy, and other agents (**Table 2**). The most frequently used agents included Pembrolizumab, Nab-paclitaxel, Atezolizumab, Camrelizumab, Paclitaxel, Carboplatin, and Durvalumab. These compounds were followed by other relevant compounds, including Nivolumab, Ipilimumab, Cyclophosphamide, Doxorubicin, Talimogene Laherparepvec, Olaparib, Gemcitabine, Capecitabine, and Apatinib. Notably, among the seven most commonly used agents, four belonged to the immunotherapy category (Pembrolizumab, Atezolizumab, Camrelizumab, and Durvalumab), while the remaining three, Nab-paclitaxel, Paclitaxel, and Carboplatin, were classified as chemotherapy drugs. The comparison of different protective drugs’ efficiency presents a significant challenge due to the variability in drug combinations used across different reported works. Moreover, not all of them measured treatment efficacy with the same clinical endpoints, further complicating direct comparisons. Despite these limitations, in **Figures 3**, **4** we have attempted to provide an overview of the relative effectiveness of several drugs for combating TNBC that will be described in more detail in the following sections.

**Table 2:** Clinical trials in TNBC focused on immunotherapy-based strategies.

Trial phase	Category	Agent	Mechanism of action	Biomarker analyzed	End point	Factor that demonstrated treatment efficacy	Effect on lymphangiogenesis	Reference
I	Immunotherapy	Atezolizumab	PD-L1 inhibitor, TI-ICs	Immune cell markers, PD-L1	*1°: safety, tolerability *2°: ORR, DOR, PFS, OS	• ORRs first (24%); second-line (6%) •DOR: 21 months • PFS: 1.4 months • OS: 17.6 months	**-**	(41)
		RNA-electroporated CAR T cells	Target cMET and initiated immune response	mRNA signals, CD3 mRNA, immune cell markers, tumor cell proliferation, T cell function	RR, PFS, OS	• PFS: 0.9 months • OS: 4.4 months	–	(42)
	Chemotherapy	Chemotherapy	Induces cytotoxic effects	**-**	PFS, OS	•PFS: 3 months • OS: 9 months	–	(43)
	Immunotherapy + Targeted therapy	Nivolumab	PD-L1 inhibitor	TILs, PD-L1, IDO	*1°: safety, tolerability *2°: CD8:FoxP3 ratio; ORR, CBR (CR, PR, SD), PFS, OS	–	–	(44)
		Ipilimumab	CTLA-4 inhibitor					
		Entinostat	HDAC inhibitor					
	Chemotherapy + Targeted therapy	Veliparib	PARP inhibitor	**-**	OR; PFS, AEs	• PFS: 8.4 months • ORR: 63%	**-**	(45)
		Carboplatin	Inhibits DNA synthesis					
		Paclitaxel	Stabilizes microtubule polymer, induces cytotoxic effects					
Ib	Immunotherapy	Talimogene laherparepvec (T-Vec)	Oncolytic Virotherapy (HSV-1)	PD-L1, LDH	*1°: DLTs *2°: ORR, OR, DOR, lesion-level response, DCR, DRR, PFS, OS, AEs	• ORR (CR/PR): 10% • DCR: 20% • PFS: 5.4 months • OS: 19.2 months	**-**	(46)
		Atezolizumab	PD-L1 inhibitor					
		Pembrolizumab	PD-L1 inhibitor	PD-L1, LDH	*1°: ORR (CR, PR) *2°: PFS, OS	• ORR: 18.5% (CR: 3.7%, PR: 14.8%) • PFS: 1.9 months • OS: 11.2 months	**-**	(47)
	Immunotherapy + Chemotherapy	Atezolizumab	PD-L1 inhibitor	PD-L1, CD8+ T, sTILs	*1°: safety, tolerability *2°: ORR, DOR, DCR, PFS, OS	• ORR: 39.4% (1CRs, 12PRs) • DOR: 9.1 months • DCR: 51.5% • PFS: 5.5 months • OS: 14.7 months	**-**	(36)
		Nab-paclitaxel	Stabilizes microtubule polymer, induces cytotoxic effects					
		Gemcitabine	Promotes apoptosis	–	*1°: safety *2°: PFS, ORR, PFS, pharmacokinetics, OS	• PFS: 9 months • ORR: 80.6% • 6-months PFS: 72.4% • DCR: 100% • DOR: 7 months	–	(48)
		Cisplatin	Induces DNA damage					
		Pucotenlimab	PD-L1 inhibitor					
	Immunotherapy + Targeted therapy	Nivolumab	PD-L1 inhibitor	TILs, MDSCs, cytokine/ chemokine, tumor and immune cell markers	*1°: MTD, RP2D, ORR *2°: PKs, TEAEs, DCR, DOR, PFS, OS	• SD: 26.7% • PF at 6 months: 21.9% • PFS: 2.6 months • OS: 6.4 months	**-**	(49)
		Mivavotinib	SYK/FLT3 inhibitor					
		Apatinib	VEGFR Inhibitor	PD-L1, BRCA mutations, VEGF	*1°: dose-limiting toxicity *2°: safety, antitumor activity pharmacokinetics	• ORR: 62% • PFS: 5 months	–	(50)
		Fuzuloparib	PARP inhibitor					
		Camrelizumab	PD-1 Inhibitor					
	Targeted therapy + others	Anlotinib	VEGFR inhibitor	ctDNA, bTMB, MSAF	*1°: ORR *2°: PFS, OS,	• ORR: 26.5% • Low MSAF, TMB had higher ORR, PFS	**-**	(51)
		Benmelstobart	PD-L1 inhibitor					
II	Immunotherapy	Pembrolizumab	PD-L1 inhibitor	PD-L1	*1°: ORR, safety *2°: DOR, DCR, PFS, OS	• ORR: 5.3% • TTR: 3.9 months • DCR: 7.6% • PFS: 2 months • OS: 9 months	–	(52)
		HER2 peptide vaccines	Induces CD4+ and CD8+ responses	HER2, immune cell markers	*1°: DFS	Trend towards clinical benefit and improved DFS	–	(53)
		Nivolumab	PD-L1 Inhibitor	PD-L1, immune cell markers	*1°: safety, adverse events *2°: efficacy	Adverse effects were mostly grade 1	**-**	(54)
		Ipilimumab	CTLA-4 Inhibitor					
		Talimogene Laherparepvec	Oncolytic Virotherapy					
		(T-VEC)	(HSV-1)					
		Mixed 19 peptide vaccine monotherapy	CTLs	IgG, C-Reactive protein, immune cell markers	*1°: OS, PFS *2°: immune response *3°: safety, tolerability	Increase IgG level CTLs positive response	–	(55)
	Chemotherapy	Taxane	Stabilizes microtubules, inhibits cell division	Immune cell markers	*1°: pCR *2°: EFS, OS, safety	• pCR: 21-41%	**-**	(56)
		Lobaplatin	Induce DNA damage					
		Anthracycline						
	Targeted therapy	Cabozantinib	MET and VEGFR2 inhibitor	Immune cell markers	*1°: CR, PR, ORR, PFS, PD, SD *2°: toxicity *3°: MET	• SD: 57%; > 15 week: 26% • CBR at 15 weeks: 34% • PFS: 2 months • Decrease in pain: 52% • ORR: 9%	–	(57)
		CR1447	Aromatase-inhibiting and androgen receptor modulating properties	Triglyceride levels	*1°: DC24 *2° DC12, CTS12, PFS24, OS, OR	• CTS12: 29.6% • PFS: 2.5 months • OS: 10.8 months	–	(58)
II	Immunotherapy + Chemotherapy	Camrelizumab	PD-1 inhibitor	PD-L1, TILs, immune cell markers	*1°: pCR *2°: bpCR, adverse events	• pCR: 65% • bpCR: 70%	–	(59)
		Non-platinum-based chemotherapy	Kills rapidly dividing cancer cells					
		Nab-paclitaxel	Stabilizes microtubule polymer, induces cytotoxic effects	PD-L1, TILs	*1°: pCR/RCB-1	• pCR: 32%	**-**	(40)
		Atezolizumab	PD-L1 inhibitor					
		Pembrolizumab	PD-L1 inhibitor	Lymphocytes, PD-L1, TILs	*1°: PFS, ORR *2°: safety, tolerability	• PFS: 4 months • OS: 15.3% • ORR: 13% • PR: 13% • SD: 33% • PD: 50% • CBR: 27%	–	(60)
		Capecitabine	Inhibits DNA synthesis					
		Nab-paclitaxel	Stabilizes microtubule polymer, induces cytotoxic effects	sTILs, iTILs, PD-L1	pCR	• pCR: 53.4%	**-**	(38)
		Durvalumab	PD-L1 inhibitor					
		Atezolizumab	PD-L1 inhibitor	PD-L1, mutations, immune gene expression	*1°: toxicity, PFS *2°: ORR, DR, DRR >6 months, CBR, OS	–	**-**	(61)
		Doxorubicin	ROS formation, induces DNA damage					
		Cyclophosphamide	DNA fragmentation					
		Carboplatin	Inhibits DNA synthesis	PD-L1, TILs, tumor DNA- and RNA-sequenced biomarkers	*1°: PFS *2°: ORR, CBR, OS	• PFS: 2.2 months • ORR: 8% • CBR: 18% • OS: 8.6 months	**-**	(62)
II	Immunotherapy + Targeted therapy	Camrelizumab	PD-L1 inhibitor	PD-L1, TILs, immune and tumor cells biomarkers	*1°: ORR *2°: DCR, DOR, CBR, TTR, PFS, OS, toxicities	• ORR: 43.3-0% • DCR: 63.3-40.0% • PFS: 3.7-1.9 months	**-**	(63)
		Apatinib	VEGFR2- inhibitor					
		Eribulin	Microtubule growth inhibition	PML, PLOD3, TILs, PD-L1, immune cell markers	*1°: ORR *2°: toxicities, DCR, CBR, PFS, 1-year overall survival	• ORR: 37% • DCR: 87% • PFS: 8.1 months	**-**	(64)
		Apatinib	VEGFR2-inhibitor					
		Camrelizumab	PD-L1 inhibitor					
		Olaparib	PARP inhibitor	mRNA, PARPi7, immune cell markers	*1°: pCR *2°: RCB, EFS, DRFS	• pCR: 27-47%	–	(65)
		Durvalumab	PD-L1 inhibitor					
	Chemotherapy + Targeted therapy	Camrelizumab	PD-L1 inhibitor	Immune cell markers, PD-L1, sTILs, somatic mutations	*1°: ORR *2°: PFS, OS, DOR, DCR, safety, tolerability	• OR: 81.3% (5/39 CRs, 34/39 PRs) • PFS: 13.6 months • TTR: 1.9 months • DOR: 14.9 months • DCR: 95.8%	**-**	(66)
		Famitinib	TRK inhibitor					
		Nab-paclitaxel	Stabilizes microtubule polymer, induces cytotoxic effects					
		Nivolumab	PD-L1 inhibitor	PD-L1, VEGF-A	*1°: ORR *2°: adverse events, disease control, PFS, OS	• OR: 10/17 • DC: 16/17 • PR: 10/17 (59%) • SD: 6/17 • PD: 1/17 • OS: 32.2 months • PFS: 7.8 months	**-**	(67)
		Bevacizumab	VEGF inhibitor					
		Paclitaxel	Stabilizes microtubule polymer, induces cytotoxic effects					
	Immunotherapy + others	Radiotherapy	Induce DNA damage	CD8 T, immune cell markers, PD-L1	OS, ORR, safety, adverse events	• ORR: 21.4% (CR: 7.1%; PR: 3.6%, SD: 10.7%) • OS: 6.6 months overall • DOT: 2.2 months overall	–	(68)
		Gene therapy	Selectively killing of virus-infected tumor cells					
		Pembrolizumab	PD-L1 inhibitor					
		Pembrolizumab	PD-L1 inhibitor	PD-L1, LDH	*1°: ORR *2°: safety, OS, PFS, irRC	• ORR: 17.6% • DOR: 4.5 months • TTR: 2.8 months • PFS: 2.6 months • OS: 11%	**-**	(69)
I/II	Immunotherapy + Chemotherapy	Durvalumab	PD-L1 inhibitor	PD-L1, TILs	*1°: pCR	• pCR: 44%	**-**	(37)
		Nab-paclitaxel	Stabilizes microtubule polymer, induces cytotoxic effects					
Ib/II	Immunotherapy + Chemotherapy	Eribulin	Microtubule growth inhibition	PD-L1, PD-L+	*1°: safety, tolerability (AE, TEAE, DLT) *2°: ORR (CR, PR) *3°: PFS, OS, DOR, CBR	• ORR: 23.4% (CR 4.8%; PR 18.6%) • SD > 8 weeks: 31.7% • DOR: 8.3 months • PFS: 4.1 months • OS: 16.1 months	–	(70)
		Pembrolizumab	PD-L1 inhibitor					
		Avelumab	PD-L1 inhibitor	PD-L1, BRCA mutations	*1°: dose-limiting toxicity, ORR *2°: safety, DOR, PFS, pharmacokinetics, immunogenicity, biomarkers	• ORR: 18% • DOR: 11 months	**-**	(71)
		Talazoparib	PARP inhibitor					
III	Chemotherapy	Epirubicin	Inhibits DNA synthesis	Immune cell markers	pCR, iDFS, DDFS, LRRFI, OS	• pCR: 50.1% • iDFS: 73-80.3% • DDFS:77.6-83.2% • OS: 82.9-88.3%	–	(72)
		Carboplatin	Inhibits DNA synthesis					
		Cyclophosphamide	DNA fragmentation					
		Doxorubicin	ROS formation, induces DNA damage					
		Paclitaxel	Stabilizes microtubule polymer, induces cytotoxic effects					
	Immunotherapy + Chemotherapy	Atezolizumab	PD-L1 inhibitor	Immune cells markers, PD-L1	*1°: OS *2°: OS (12- and 18 months), PFS, ORR, DOR, CBR, PROs, safety, pharmacokinetics	**-**	**-**	(73)
		Gemcitabine	Promotes apoptosis					
		Capecitabine	Inhibits DNA synthesis					
III	Immunotherapy + Chemotherapy	Nab-paclitaxel	Stabilizes microtubule polymer, induces cytotoxic effects	Distribution of PD-L1-positive immune cells	*1°: PFS, OS *2°: OR, DOR	• OS: 7 months • PFS: 2.2 months	–	(39)
		Atezolizumab	PD-L1 inhibitor					
		Pembrolizumab	PD-L1 inhibitor	PD-L1, immune cell markers, tumor RNA	ORR, DOR, PFS, OS, safety, tolerability	• ORR: 48% • DOR: 6 months • PFS: 6 months • OS: 13 months	–	(74)
		Carboplatin	PD-L1 inhibitor					
		Nab-paclitaxel	Induces cytotoxic effects					
	Immunotherapy + others	Datopotamab deruxtecan	Induces DNA damage/TROP2 antibody	–	*1°: iDFS *2°: safety, DDFS, OS, TTD	–	–	(75)
		Durvalumab	PD-L1 inhibitor					

AEs, Adverse Events; bpCR, Breast Pathologic Complete Response; CBR, Clinical Benefit Rate; CR, Complete Response; ctDNA, Circulating Tumor DNA; CTS12, Clinical Trial Subscore 12; DCR, Disease Control Rate; DDFS, Distant Disease-Free Survival; DFS, Disease-Free Survival; DOR, Duration of Response; DRR, Durable Response Rate; EFS, Event-Free Survival; iDFS, Invasive Disease-Free Survival; irRC, Immune-Related Response Criteria; MET, Metastasis; MSAF, Maximum Somatic Allele Frequency; MTD, Maximum Tolerated Dose; OFS, Overall Free Survival; ORR, Overall Response Rate; OS, Overall Survival; CpR, Pathological Complete Response; PFS24, 24-Month Progression-Free Survival; PFS, Progression-Free Survival; PKs, Pharmacokinetics; PR, Partial Response; PROs, Patient-Reported Outcomes; RP2D, Recommended Phase 2 Dose; SD, Stable Disease; STILs, Stromal Tumor-Infiltrating Lymphocytes; TEAEs, Treatment-Emergent Adverse Events; TMB, Tumor Mutational Burden; TTD, Time to Deterioration; TTR, Time to Response.

#### 3.2.1 Immunotherapy agents

##### 3.2.1.1 Pembrolizumab

Pembrolizumab is a potent humanized monoclonal antibody of the IgG4-kappa subclass that selectively targets the PD-1 receptor on human cells. It was developed by grafting the variable region sequences of a mouse anti-human PD-1 antibody onto a human IgG4-k isotype framework containing a stabilizing S228P Fc mutation (76, 77). As a PD-1 inhibitor, Pembrolizumab blocks the immune suppression signals that tumor cells use to evade destruction by the immune system. By preventing PD-1 from interacting with its ligands, PD-L1 and PD-L2, it facilitates the activation and proliferation of cytotoxic T lymphocytes in the TME, leading to a more effective anti-tumor immune response. It has demonstrated robust and durable efficacy in various diseases (76, 78, 79).

This antibody has a well-characterized safety profile and has been approved for the treatment of multiple cancer types, including melanoma, non-small-cell lung cancer (NSCLC), classical Hodgkin’s lymphoma, metastatic urothelial carcinoma, head and neck squamous cell carcinoma, gastric cancer, and TNBC, among others (52, 77, 78). The recommended dosage varies depending on the treatment regimen and cancer type but is generally administered as an intravenous infusion at a dose of 200 mg every three weeks (76, 79). The most common side effects of Pembrolizumab include fatigue, nausea, pruritus, rash, diarrhea, and loss of appetite. Due to its mechanism of action, it may also induce immune-related adverse reactions such as pneumonitis, colitis, and hepatitis (76, 78).

Regarding TNBC treatment, Pembrolizumab has shown promising efficacy (47, 52, 69, 70, 74, 80). In terms of OS, monotherapy achieved an average of 10.1 months, whereas combination therapies extended OS to 12.4 months. When Pembrolizumab was used alone in the therapy, PFS was reported to be less than 2 months. However, when combined with two or three other agents, PFS improved, ranging between 2.6–6 months, regardless of the specific agents combined. Additionally, the overall response rate (ORR) averaged 11.9% with monotherapy but increased to 24.7% when it was combined with other agents. Among the evaluated combinations, Pembrolizumab with Carboplatin and Nab-placlitaxel emerged as the most effective for TNBC treatment, followed by Pembrolizumab with Eribulin (**Figure 3a**).

##### 3.2.1.2 Atezolizumab

Atezolizumab is a humanized monoclonal antibody of the IgG1 subclass that selectively targets PD-L1. By binding to PD-L1, it prevents its interaction with PD-1 or CD80 (B7-1) receptors, thereby restoring T-cell activation and enhancing the immune response against tumor cells. Unlike PD-1 inhibitors, which act directly on T-cells, Atezolizumab disrupts the immunosuppressive signal at the level of tumor and antigen-presenting cells, allowing for a broader modulation of the immune system (78, 81).

Atezolizumab is generally well tolerated and has good clinical activity in multiple cancers, including metastatic TNBC, renal cell carcinoma, and bladder transitional cell carcinoma (78). It is commonly administered as an intravenous infusion at a standard dose of 1,200 mg every three weeks although dosing can vary on the treatment regimen and cancer type (81). The most common side effects of Atezolizumab include fatigue, nausea, decreased appetite, cough, and rash. It can also cause immune-related adverse events, such as pneumonitis, colitis, hepatitis, endocrinopathies, and myocarditis (78, 82).

Whitin the clinical trials that employed immunotherapy agents, Atezolizumab was the second most frequently used (36, 39–41, 46, 62). Among the evaluated treatment combinations, Atezolizumab plus Nab-paclitaxel demonstrated the highest efficacy in terms of PFS and ORR. However, when OS was considered, Atezolizumab with Talimogene Laherparepvec yielded superior outcomes (**Figure 3b**).

##### 3.2.1.3 Camrelizumab

Camrelizumab is a fully humanized IgG4-kappa monoclonal antibody that targets PD-1 (63). Upon administration, it blocks the interaction between PD-1 and its ligands PD-L1 and PD-L2, which are overexpressed on certain cancer cells and primarily found on antigen presenting cells, respectively. This blockade prevents PD-1 activation and its downstream signaling pathways and restores immune function through the activation of cytotoxic T lymphocytes and cell-mediated immune responses against tumor cells. Activated PD-1 negatively regulates T-cell activation and plays a key role in tumor evasion from host immunity (83).

Although Camrelizumab has been commercially available only since 2019, it has demonstrated promising efficacy and an acceptable tolerance in several kinds of solid tumors, including esophageal, hepatocellular, and renal cell carcinoma, as well as NSCLC and TNBC (83, 84). The recommended dose is 200 mg administered intravenously every two weeks (83). Common side effects include anemia, fever, fatigue, hypothyroidism, proteinuria and cough, and immune-related adverse events such as pneumonitis and colitis (83).

In recent years, the role of Camrelizumab in TNBC is gaining attention (50, 59, 63, 66, 85). Camrelizumab combined with Nab-paclitaxel and Famitinib demonstrated the highest efficacy, achieving a PFS of 13.6 months. Additionally, the combination of Camrelizumab with Apatinib and Fuzuloparib yielded the highest ORR at 63% (**Figure 3c**).

##### 3.2.1.4 Durvalumab

Durvalumab is an IgG1-kappa monoclonal antibody that has high affinity binding to PD-L1, blocking its interaction with PD-1 and CD80 (B7-1) receptors on T-cells. This blockade prevents tumor immune evasion and enhances anti-tumor immune responses (78, 86).

Durvalumab is primarily approved for the treatment of platinum-resistant, advanced, or metastatic urothelial carcinoma in PD-L1-positive patients, as well as NSCLC. Additionally, it is under investigation for other solid tumors, including TNBC (78). The standard dosing regimen is 10 mg/kg, administered intravenously every two weeks (87), although this may vary depending on the indication and specific treatment protocols. The most common adverse events are fatigue, diarrhea, and decreased appetite (86).

The effectiveness of Durvalumab has been evaluated in TNBC through three clinical studies reporting pCR (37, 38, 65). Its combination with Nab-paclitaxel resulted in higher pCR rates (> 44%) compared to its combination with Paclitaxel and Olaparib (< 37%) (**Figure 3d**).

#### 3.2.2 Chemotherapy agents

##### 3.2.2.1 Nab-paclitaxel

Nanoparticle albumin-bound paclitaxel, commonly known as Nab-paclitaxel, is an advanced formulation of Paclitaxel designed to enhance drug delivery and reduce toxicity associated with traditional solvent-based formulations. Unlike conventional Paclitaxel, Nab-paclitaxel does not require solvents such as polyoxyethylated castor oil and ethanol, which have been associated to toxic responses, including hypersensitivity reactions and prolonged sensory neuropathy (88, 89). This albumin-bound formulation enhances tumor penetration, leading to greater antitumor activity while minimizing toxicity compared to solvent-based Paclitaxel. Nab-paclitaxel exerts its antitumor effects by inhibiting the depolymerization of microtubules, a process necessary for normal cell division, disrupting cell division, and leading to apoptosis in rapidly proliferating cancer cells (88, 89).

Nab-paclitaxel is approved for the treatment of multiple cancer types, including ovarian cancer, metastatic breast cancer, NSCLC, and pancreatic cancer (90). The standard dosage for metastatic breast cancer is 260 mg/m², administered as an intravenous infusion every three weeks, although dosing regimens vary depending on the type of cancer and individual patient factors (89). Common side effects of Nab-paclitaxel include neutropenia, anemia, sensory neuropathy, myalgia, and nausea (88, 90).

Regarding TNBC treatment, Nab-paclitaxel was tested in three studies in combination with Atezolizumab. However, differing results were observed for the same efficiency variables: OS ranged from 7 to 14.7 months, while PFS varied between 2.2 and 5.5% (36, 39, 40). The combination of Nab-paclitaxel and Durvalumab was also evaluated in two studies, showing comparable results in terms of pCR, ranging from 44 to 53.4% (37, 38). Other combinations have demonstrated promising outcomes in terms of ORR (66, 74) (**Figure 4a**).

##### 3.2.2.2 Paclitaxel

Paclitaxel is a natural product extracted as a white powder from the bark of a Pacific yew tree (*Taxus brevifolia*). As a member of the taxane family, Paclitaxel exhibits a strong affinity for microtubules, thus impeding their depolymerization process. Microtubules are responsible for mitotic spindle formation throughout cell division and are needed for cell structure maintenance and motility of the cell with its intracellular cytoplasm. As a result, Paclitaxel limits the development of cancer cells by causing a stop in the cell cycle phases, specifically at the G2/M phase (91).

Paclitaxel is widely employed in the treatment of breast, ovarian, lung, esophageal, gastric, pancreatic, and head and neck cancers (91). The standard dose ranges from 135 to 175 mg/m², administered intravenously over three hours every three weeks, depending on the specific treatment regimen. Paclitaxel can cause a range of adverse effects. The most prevalent adverse effects of Paclitaxel use are alopecia, nausea and vomiting, mucositis, neutropenia, leukopenia, anemia, hypersensitivity reactions, arthralgia, myalgia, and weakness (92).

In the clinical trials reviewed, Paclitaxel was evaluated in combination with agents other than Nab-paclitaxel, limiting direct comparisons between the two compounds (45, 65, 67, 72). Specifically, the combination of Paclitaxel with Carboplatin, Epirubicin, Cyclophosphamide, and Doxorubicin showed superior efficacy in achieving pCR compared to Paclitaxel combined with Durvalumab and Olaparib, with pCR rates of 50.1 and 37%, respectively (**Figure 4b**).

##### 3.2.2.3 Carboplatin

Carboplatin is a platinum-based chemotherapeutic agent, structurally similar to cisplatin, but with a more favorable toxicity profile. The drug exerts its effects primarily by binding to DNA, forming platinum-DNA adducts that inhibit DNA replication and transcription, inducing cell death. The formation of these adducts interferes with several cellular signaling pathways, triggering apoptosis or necrosis in cancer cells. However, resistance to platinum-based therapies, including Carboplatin, remains a significant clinical challenge, as the mechanisms underlying this resistance are multifactorial and not fully understood (93).

This chemotherapeutic drug is widely used for cancer of ovarian, lung, head, and neck (94). Carboplatin is administered intravenously at 240 mg/m^2^ every three to four weeks. Although Carboplatin is less toxic than cisplatin, it still carries several potential side effects, including anemia, leukopenia, thrombocytopenia, nausea, vomiting, and fatigue.

The effectiveness of Carboplatin in TNBC has been widely studied (45, 62, 72, 74). Notably, PFS ORR were improved when Carboplatin was combined with two additional drugs in the treatment. In contrast, the inclusion of only one drug resulted in decreased PFS and ORR. Regarding OS, only two of the studies reported this value. These findings were consistent with other measured indicators, showing longer survival times in patients receiving therapies incorporating three agents compared to those treated with two-agent regimens (**Figure 4c**).

These therapeutic strategies and agents have demonstrated promising results in specific TNBC patient subgroups. However, there is a critical need to develop more effective therapies and further explore the potential of combining immunotherapy, chemotherapy, and targeted agents to improve clinical outcomes. Future research could be guided by a dual approach that integrates immune modulation and lymphangiogenesis, offering a promising path towards more precise and effective treatments for this aggressive breast cancer subtype.

### 3.3 Modulation of lymphangiogenesis in triple-negative breast cancer as a promising approach to limit progression and enhance immunity

**Figure 5:**
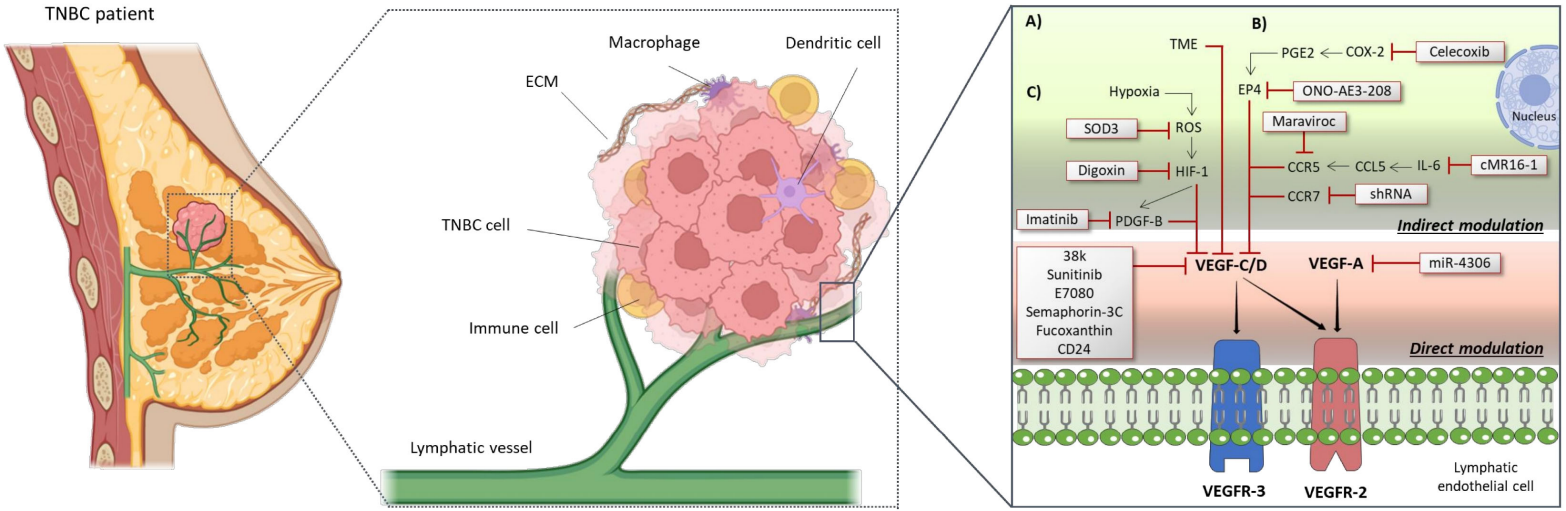
Strategies (direct and indirect: **(a)** TME; **(b)** Inflammatory pathways; **(c)** Hypoxia) for modulating lymphangiogenesis in triple-negative breast cancer. CCL5, C-C motif chemokine ligand 5; CCR, C-C chemokine receptor; COX-2, Cyclo-oxygenase 2; ECM, Extracellular matrix; EP4, Prostaglandin E2 receptor 4; HIF-1, Hypoxia-inducible factor 1; IL-6, Interleukin 6; miR-4306, MicroRNA 4306; PDGF-B, Platelet-derived growth factor B; PGE2, Prostaglandin E2; ROS, Reactive oxygen species; TME, Tumor microenvironment; TNBC, Triple-negative breast cancer; VEGF, Vascular endothelial growth factor; VEGFR, Vascular endothelial growth factor receptor.

We identified 19 studies focused on modulating lymphangio-genesis as a therapeutic approach to limit the progression of TNBC. Collectively, these studies showed that disrupting the formation or function of lymphatic vessels, by employing a wide range of therapeutic strategies and agents, can interfere with the metastatic cascade, particularly by limiting the spread of tumor cells to regional LNs and distant organs (95–113). In addition, some of these studies highlighted that modulation of the lymphangiogenic process not only impedes physical tumor dissemination but also alters the TME, enhancing anti-tumor immune responses and reducing immunosuppression (**Figure 5**). These findings underscore the potential of therapies targeting lymphatic vessels as key component in the development of innovative treatment strategies aimed at improving clinical outcomes for TNBC patients (95–113).

#### 3.3.1 Direct modulation of tumor-associated lymphangiogenesis via blockade of VEGF-mediated signaling

Lymphangiogenesis is mainly regulated by the vascular endothelial growth factor receptor-3 (VEGFR-3), a tyrosine kinase receptor expressed primarily on lymphatic endothelial cells (LECs). Several studies have revealed VEGFR-3 overexpression in breast cancer, with evidence supporting its role as a promising therapeutic target in TNBC via lymphangiogenesis inhibition (109–115).

A VEGFR-3 inhibitor, N-(4-Chloro-3-(trifluoromethyl)phenyl)-4-(6-(4-(4-methylpiperazin-1-yl)phenyl)thieno[2,3-d]pyrimidin-4-yl)piperazine-1-carboxamide (compound 38k), significantly suppressed lymphatic vessel formation and invasion in LECs and breast cancer cells. It exerted both anti-proliferative and anti-migration activities by inhibiting the VEGFR-3 signaling pathway (111). Similarly, Sunitinib, a multi-kinase inhibitor, blocked VEGFRs activity attenuating the cellular functions of LECs (proliferation, migration, and tube formation). In addition, it suppressed tumor-induced lymphangiogenesis and LN metastasis (110). Another potent inhibitor of VEGFRs, E7080, decreased lymphatic vessel density in breast cancer cells and LNs after resection of the primary tumor (112). Semaphorin-3C strongly inhibited the proliferation of LECs by reducing VEGF-C induced signal transduction. It also reduced lymphatic vessel density within tumors and suppressed metastatic spread (113).

Fucoxanthin is a material found in brown algae with a wide variety of pharmacological functions, including anti-tumor and anti‐inflammatory effects. Fucoxanthin inhibited proliferation, migration, and formation of tube‐like structures in LECs by decreasing levels of VEGF‐C and VEGFR‐3, while reducing lymphangiogenesis and characteristics of the tumor such as its weight and volume (114). On the other hand, it was determined that the expression of the CD24 glycoprotein was elevated in lung and LN metastatic TNBC cells. Its depletion, through VEGF-C and VEGF-A downregulation, inhibited primary tumor growth and LN and lung metastasis (109). Moreover, upregulation of the microRNA miR-4306 suppressed TNBC cell proliferation, migration, and invasion and inhibited lymphangiogenesis in *in vitro* assays. In *in vivo* models, its overexpression significantly reduced TNBC growth as well as lung and LN metastasis, primary by targeting VEGF-A (115) (**Figure 5**).

#### 3.3.2 Strategies for the indirect modulation of lymphangiogenesis

##### 3.3.2.1 Impact of the TME on lymphangiogenesis

It is known that LECs are an essential part of the TME and that tumor-associated lymphangiogenesis can be indirectly induced by TME-derived lymphangiogenic factors (107). Consequently, the TME can be conditioned to function as a pro-metastatic niche due to the diverse secreted factors, suggesting that it is not a static entity and can be strategically modulated to counteract pro-lymphangiogenic signals (102–108).

Using a three-dimensional co-culture model with LEC and TNBC cells, the interactions between tumor cells and the lymphatic system were demonstrated (104). These interactions were mediated by factors released by tumor cells that promoted the activation of LECs, stimulated the lymphangiogenic process and contributed to metastatic progression (104). Similarly, a tumor-lymphatic microfluidic model revealed changes in genes related to vessel growth, permeability, metabolism, hypoxia, and apoptosis in LECs co-cultured with TNBC cells (102). Changes in inflammation-associated genes, such as adhesion molecules and chemokines, were also observed in tumor-associated LECs. In particular, it has been observed that in both *in vitro* and *in vivo* assays, blockade of the adhesion molecule VCAM-1 reduced tumor-induced lymphatic permeability and lymphatic invasion (103). The communication between endothelial and tumor cells could be disrupted by a CD47-blocking antibody, leading to reduced tumor-induced lymphangiogenesis and impaired metastatic dissemination (105). Another study investigated the role of a long non-coding RNA, HUMT, highly upregulated in metastatic TNBC-lymph node and with an important role on cancer cell proliferation and metastasis. It was shown that HUMT, which exerted its function by activating the Akt/mTOR/VEGF-C pathway, promoted proliferation, lymphangiogenesis, and LN metastasis in TNBC (108). It was also demonstrated that LECs can be conditioned by TNBC cells to accelerate metastasis. LECs within the lungs and LNs, conditioned by tumor-secreted factors expressed CCL5, a protein that induced lymphangiogenesis and facilitated metastasis (107). Similarly, an *in vivo* assay demonstrated that the LNs, primary tumors, and lungs of mice treated with tumor-conditioned media enhanced lymphangiogenesis. These animals also showed metastatic dissemination to the abdomen from the primary injection site, which would generally enhance metastasis to other organs (106) (**Figure 5a**).

Taking altogether we can conclude that the TNBC microenvironment has an important impact on lymphangiogenesis and the LEC behavior.

##### 3.3.2.2 Targeting inflammatory pathways to modulate lymphangiogenesis

Lymphangiogenesis in TNBC can also be modulated through the inhibition of inflammatory pathways (116–118). As such, the secretion of the CCL5 protein by LECs induced lymphangiogenesis and facilitated subsequent lung metastasis (116). IL-6, a cytokine with complex roles in inflammation and metabolic disease secreted by TNBC cells, was a key factor in upregulating CCL5 expression in LECs. Accordingly, the use of Maraviroc, a CCR5 inhibitor, and cMR16-1, a murine surrogate of the anti-IL-6 receptor antibody, demonstrated that simultaneous blockade of CCR5 and IL-6 receptor signaling strongly suppresses tumor growth and significantly inhibits metastasis in a TNBC model using athymic nude mice (116). CCR7, a member of the C-C chemokine receptor family, is typically expressed in immune cells. Its knockdown via shRNA was found to reduce proliferation, migration, and invasion of TNBC cells *in vitro*, as well as to reduce the distant metastasis in an orthotopic mouse model (117). On the other hand, it was observed that the overexpression of cyclo-oxygenase (COX)-2, an inflammation-associated enzyme, is one of the major determinants of tumor progression and metastasis in a wide variety of cancers including breast cancer (118). Celecoxib and ONO-AE3-208, inhibitors of COX-2 and the EP4 receptor respectively, have demonstrated efficacy in reducing tumor growth, lymphangiogenesis, and metastasis to LNs and lungs in a preclinical model of breast cancer (118) (**Figure 5b**).

##### 3.3.2.3 Modulation of lymphangiogenesis through hypoxia induced signaling

Another strategy to indirectly modulate lymphangiogenesis is through hypoxia. Severe hypoxia in breast cancer is associated with a significantly increased risk of metastasis and patient mortality. Hypoxia has been shown to regulate lymphangiogenesis by promoting the overexpression of specific lymphangiogenic factors such as VEGF-C (119, 120). One of the major mechanisms by which hypoxia promotes metastasis in TNBC is through the activation of hypoxia-inducible factor 1 (HIF-1). HIF-1 directly transactivates the gene encoding platelet-derived growth factor B (PDGF-B), which exerts proliferative and chemotactic effects on LECs, thereby promoting lymphatic metastasis. In a human breast cancer orthograft mouse model, treatment with the HIF-1 inhibitor Digoxin or the tyrosine kinase inhibitor Imatinib significantly reduced peritumoral lymphangiogenesis as well as LNs metastasis (119). In turn, hypoxia also increases the production of reactive oxygen species (ROS) in tumor cells. ROS accumulation activated signaling pathways that increased VEGF-C expression, possibly through the regulation of HIF-1. Authors suggested that the inhibition of VEGF-C with superoxide dismutase 3 (SOD3), an antioxidant enzyme, could be an effective strategy to counteract breast cancer progression in hypoxic environments by simultaneously blocking lymphangiogenesis and antioxidant adaptations of tumor cells (120) (**Figure 5c**).

Therefore, it has been demonstrated that targeting lymphangiogenesis has emerged as a promising therapeutic strategy not only to limit tumor progression and metastasis in TNBC, but also to modulate the TME in favor of antitumor immunity. So, this approach could have the potential to complement existing therapies and ultimately improve patient outcomes.

## 4 Discussion

Triple-negative breast cancer is the only subtype of breast cancer that lacks specific targeted therapies, making it particularly challenging to treat effectively. Its lower survival rate is attributed to several aspects of the disease, including its higher genomic heterogeneity, elevated tumor grade and mitotic index, mutations in the p53 and BRCA1 genes, and increased lymphatic dissemination, all contributing to its aggressive nature. Due to the lack of specific molecular targets and significant heterogeneity, chemotherapy remains the primary treatment for TNBC, with targeted therapies still under investigation and offering limited results (22, 121, 122).

In recent years, immunotherapy has emerged as a promising treatment strategy that activates the immune system to combat cancer cells. The most effective immunotherapeutic agents are the immune checkpoint inhibitors (ICIs), which enhance anti-tumor immunity by targeting the PD-L1 ligand on tumor cells, and have been reported to be promising therapeutic agents for TNBC treatment (121). Atezolizumab, Avelumab, Benmelstobart, Camrelizumab, Durvalumab, Nivolumab, Pembrolizumab, and Pucotenlimab are ICIs that have been used in the treatment of TNBC, being Pembrolizumab, Atezolizumab, Camrelizumab, and Durvalumab the most popular (**Table 2**). These PD-1/PD-L1 inhibitors have demonstrated significant efficacy in TNBC patients (**Figure 3**). Studies confirmed that monotherapy of Pembrolizumab offered sustained anti-tumor activity in patients with advanced PD-L1-positive TNBC (47, 52). Atezolizumab was also explored as ICI monotherapy in metastatic TNBC. Phase I clinical trial showed that Atezolizumab had good safety and object response rate in TNBC patients (41). The combination of ICIs with other treatments is emerging as a novel strategy for improving outcomes in TNBC. They can be combined with conventional treatments such as chemotherapy, radiotherapy, or targeted therapies (123). A trial showed a significant improvement in progression-free survival using Atezolizumab plus Nab-paclitaxel instead of placebo plus Nab-paclitaxel (39). The combination of Pembrolizumab and radiotherapy was also safe and showed promising activity in patients with poor-prognosis and metastatic TNBC (69). A phase II clinical trial combined Camrelizumab with Apatinib, a VEGFR inhibitor, in patients with advanced TNBC. This trial demonstrated that the combination of these two drugs was safe and showed a superior clinical response to a single drug application (63).

Despite the growing interest in immunotherapy as treatment for TNBC, no clinical studies to date have specifically evaluated the impact of combination immunotherapy with agents targeting lymphangiogenesis. Most clinical trials have primarily focused on assessing the direct effects of therapeutic agents on TNBC progression, tumor reduction, and overall survival improvement, without considering their potential impact on the lymphatic system or the remodeling of lymphatic vessels present in the TNBC microenvironment (**Table 2**). One of the key reasons for developing therapeutic agents targeting lymphangiogenesis is its central role in facilitating tumor metastasis. Tumor cells, including those from breast cancer, take advantage of the cell transport capabilities of the lymphatic system due to their unique structural features and preferentially disseminate through the lymphatic vessels to reach regional LNs and distant organs, thereby facilitating the metastatic process (18, 95). In addition, the slower flow dynamics within lymphatic system further protect disseminating cells from mechanical stress, enhancing their survival during transit (18, 95–98). Once inside the lymphatic vessels, breast cancer cells are transported to regional LNs, which frequently serve as the initial site of metastatic colonization. Upon arrival, these tumor cells undergo a series of metabolic changes to adapt to the LNs microenvironment. These adaptations facilitate immune evasion, supporting tumor cell survival and proliferation. Beyond acting as a reservoir for metastatic tumor growth, LNs also serve as key hubs for secondary dissemination. From this site, breast cancer cells can enter the systemic circulation, promoting further metastasis to distant organs. Breast cancer cells possess an organ-specific pattern of dissemination and metastasize to bone (47−60%), and subsequently to the liver (19-20%), lung (16-34%), and brain (10-16%) (18, 95–98).

The infiltration of tumor cells into lymphatic vessels and regional LNs actively promotes lymphangiogenesis (99). Interestingly, modulating lymphangiogenesis not only impacts the metastatic potential of breast cancer but also affect the TME, altering immune cell dynamics. These alterations include a modification in the ratio of immune cells and release of multiple immune inhibitory and reactive cytokines (100). Importantly, TNBC is typically associated with a high density of TILs, associated with the tumor’s immune escape mechanisms. Additionally, TNBC cells can evade immune surveillance by down-regulating NK cell receptors or secreting inhibitory factors (101).

Regarding the molecular signaling pathways promoting lymphangiogenesis, the vascular endothelial growth factor (VEGF)-C, and -D play a critical role in lymphangiogenesis by activating tyrosine kinase receptors, primarily VEGFR-3. Since the VEGF-C/D-VEGFR-3 signaling axis is a crucial and highly specific pathway for inducing lymphangiogenesis, and sufficient preclinical evidence reveals that blocking VEGF-C/D or VEGFR-3 significantly inhibits lymphangiogenesis and suppresses tumor LN metastasis, it is not surprising that different drugs have been designed to target this pathway, aiming to inhibit tumor-associated lymphangiogenesis and treat metastatic cancer (124). However, lymphangiogenesis is also regulated by additional pro-lymphangiogenic mediators within the TME that can represent alternative therapeutic targets. As mentioned above, lymphangiogenesis in TNBC can be modulated by various agents that directly or indirectly target VEGFR-3 signaling and its associated pathways (**Figure 5**). Agents such as compound 38k, Sunitinib, and E7080, significantly suppressed lymphatic vessel formation, LEC activity and metastasis through direct inhibition of VEGFR-3 signaling (110–112). These findings highlight the central role of the VEGF-C/VEGFR-3 axis as a key therapeutic target in tumor-associated lymphangiogenesis.

Interestingly, emerging preclinical evidence suggests that certain systemic therapies may also modulate lymphatic vessel biology. For example, platinum-based chemotherapy has been shown to induce VEGFR3-dependent lymphangiogenesis, an effect that can be reversed by anti-VEGFR3 treatment (125), underscoring the dynamic and context-dependent effects of conventional therapies on the lymphatic vasculature.

Beyond direct targeting of VEGF signaling, lymphangiogenesis is also regulated by inflammatory and hypoxia-related pathways, further increasing the complexity of its modulation within the TME. In this context, several chemotherapeutic and immunotherapeutic agents may indirectly influence lymphatic vessel formation and function. Taxanes and platinum-based agents, for instance, can alter inflammatory signaling and VEGF family expression, thereby impacting LEC behavior. Similarly, immune checkpoint inhibitors, such as anti-PD-1/PD-L1 antibodies, may modulate lymphatic vessel function by reshaping immune cell trafficking and cytokine gradients within the TME. Although these indirect effects remain incompletely understood, they suggest that standard therapies may exert previously underappreciated roles in regulating tumor-associated lymphangiogenesis, opening new avenues for combinatorial therapeutic strategies.

Anti-inflammatory strategies, including COX-2 and IL-6 pathway inhibitors, reduced lymphangiogenesis and LN metastasis by disrupting pro-lymphangiogenic inflammatory signals (116, 118). Lymphangiogenesis can also be indirectly inhibited through hypoxia, with agents such as HIF-1 inhibitors or antioxidants, that have been shown to reduce lymphatic vessel density and metastatic potential (119, 120). In addition, targeting the TME and its associated molecular pathways also contributes to the modulation of lymphangiogenesis, offering a multifaceted approach to counteract metastasis (102–108).

Recently, the role of lymphangiogenesis in cancer immuno-therapy has garnered attention with studies suggesting that lymphangiogenesis modulation could be clinically useful in combination with immunotherapy (124). In melanoma, it has been reported that tumor lymphangiogenesis enhanced the infiltration of T-cells into the tumor, thereby potentiating the efficacy of immunotherapy by promoting stronger immune responses (126). In addition, both human and murine melanoma models have demonstrated that lymphatic vessels play a crucial role in regulating the immune microenvironment (127). A deficiency in lymphatic vessels impaired the infiltration and activation of CD8-positive T-cells, suggesting that the immunotherapy response was dependent on the sprouting of lymphatic vessels (127). Following this approach, similar strategies could be applied to TNBC, where modulating lymphangiogenesis may enhance immune responses, thus offering hope for better outcomes and combined therapies in TNBC patients.

Although immunotherapy has significantly reshaped the therapeutic landscape of TNBC, non-immune targeted therapies continue to play an important role in its clinical management (128). Given the marked molecular heterogeneity and aggressive clinical behavior of TNBC, multiple signaling pathways involved in tumor proliferation, cell cycle regulation, DNA damage response, and survival have been investigated as potential therapeutic targets (129). Accordingly, several molecularly targeted agents have been developed and evaluated in clinical studies, either as monotherapy or in combination strategies, to improve treatment outcomes. These include tyrosine kinase inhibitors targeting EGFR and VEGFR, serine/threonine kinase inhibitors affecting the PI3K/AKT/mTOR axis, CDK4/6 inhibitors, and agents targeting the DNA damage response such as ATR, WEE1, and CHK1 inhibitors. In addition, PARP inhibitors, proteasome inhibitors, and epigenetic modulators such as histone deacetylase (HDAC) inhibitors have been explored in TNBC, including in combination with chemotherapy and ICIs to enhance therapeutic efficacy and overcome resistance mechanism. Despite these promising advances, the clinical benefits of many targeted therapies remain limited by the marked molecular heterogeneity of TNBC and the lack of reliable predictive biomarkers for appropriate patient stratification (130).

In the current context, comprehensive molecular profiling of the TNBC microenvironment, including both immune and lymphatic components, may improve patient stratification and guide the selection of more effective therapeutic strategies. Emerging technologies such as single-cell and spatial transcriptomics, together with advanced TME modelling, are enhancing our understanding of the complex interactions between tumor cells, immune populations, and lymphatic endothelial cells (131–134). These advances are expected to enable the identification of novel therapeutic targets and support the development of more effective combination approaches that integrate immunotherapy with the modulation of tumor-associated lymphangiogenesis.

## 5 Conclusion

The treatment of TNBC remains challenging due to its aggressive nature and high metastatic potential. Although lymphangiogenesis plays a crucial role in TNBC and its modulation has shown to be effective in treating this cancer subtype, the impact of combination immunotherapy approaches targeting lymphangiogenesis has not been explored yet. This systematic review highlights the need for further investigation into how affecting lymphangiogenesis with specific agents could enhance immune responses in TNBC patients treated with immunotherapy, achieving more durable responses. Ultimately, integrating therapies that focus on lymphangiogenesis could not only improve the effectiveness of immunotherapy but also provide new insights for the clinical management of this cancer subtype.
